# Hard X‐Ray Nanoholotomography: Large‐Scale, Label‐Free, 3D Neuroimaging beyond Optical Limit

**DOI:** 10.1002/advs.201700694

**Published:** 2018-03-30

**Authors:** Anna Khimchenko, Christos Bikis, Alexandra Pacureanu, Simone E. Hieber, Peter Thalmann, Hans Deyhle, Gabriel Schweighauser, Jürgen Hench, Stephan Frank, Magdalena Müller‐Gerbl, Georg Schulz, Peter Cloetens, Bert Müller

**Affiliations:** ^1^ Biomaterials Science Center (BMC) Department of Biomedical Engineering University of Basel 4123 Allschwil Switzerland; ^2^ ID16A‐NI Nano‐Imaging Beamline European Synchrotron Radiation Facility (ESRF) 38043 Grenoble France; ^3^ Institute of Pathology Department of Neuropathology Basel University Hospital 4056 Basel Switzerland; ^4^ Musculoskeletal Research Group Department of Biomedicine University of Basel 4056 Basel Switzerland

**Keywords:** cerebellum, hierarchical imaging, human brain, neocortexes, neuroimaging, segmentation

## Abstract

There have been great efforts on the nanoscale 3D probing of brain tissues to image subcellular morphologies. However, limitations in terms of tissue coverage, anisotropic resolution, stain dependence, and complex sample preparation all hinder achieving a better understanding of the human brain functioning in the subcellular context. Herein, X‐ray nanoholotomography is introduced as an emerging synchrotron radiation‐based technology for large‐scale, label‐free, direct imaging with isotropic voxel sizes down to 25 nm, exhibiting a spatial resolution down to 88 nm. The procedure is nondestructive as it does not require physical slicing. Hence, it allows subsequent imaging by complementary techniques, including histology. The feasibility of this 3D imaging approach is demonstrated on human cerebellum and neocortex specimens derived from paraffin‐embedded tissue blocks. The obtained results are compared to hematoxylin and eosin stained histological sections and showcase the ability for rapid hierarchical neuroimaging and automatic rebuilding of the neuronal architecture at the level of a single cell nucleolus. The findings indicate that nanoholotomography can complement microscopy not only by large isotropic volumetric data but also by morphological details on the sub‐100 nm level, addressing many of the present challenges in brain tissue characterization and probably becoming an important tool in nanoanatomy.

## 1 Introduction

In our aging society, incidence and prevalence of brain disorders are rapidly increasing, with almost one‐third of disabilities being attributed to brain malfunction.^1^ Micro‐ and nanomorphology of the neuronal network is tightly linked with the brain's functionality. This has sparked significant interest and efforts aimed at uncovering hierarchically organized neuronal structures.^2^ Currently available imaging methodologies, however, are limited in their 3D representation of large specimens in a time‐efficient manner with sufficient nanoscale isotropic resolution while preserving the biological context.

Although the sample preparation remains a complicated and error‐prone process, substantial effort has been devoted to revealing the 3D microstructure of brain tissue ex vivo, with or without the requirement for serial sectioning.^3^ Despite continuous advances, the 3D analysis of subcellular structures based on 2D histological sections in combination with microscopy is limited by sectioning‐ or staining‐related artefacts, and may lead to misinterpretation of the results due to the lack of volumetric information.^4^ Serial sectioning or optical‐ablative methods, in combination with 3D image reconstruction, are labor‐ and computation‐intensive, requiring acquisition times of days to obtain single‐cell resolution,^5^ and still hinder continuous observations.^6^ Technically demanding protocols for rendering tissue optically transparent, for example, CLARITY,^5^, ^7^ PACT,^8^ or CUBIC,^6^ remain time‐consuming and, subsequently to tissue clearing, powerful histology can only partially be applied, due to stain or antibody binding reduction or tissue degradation.

By overcoming optical microscopy limitations, advanced nanoscale X‐ray imaging techniques, such as ptychographic X‐ray computed tomography,^9^ small‐angle X‐ray scattering computed tomography,^10^ X‐ray microscopy,^11^ or transmission soft X‐ray tomography,^12^ can enhance the 3D visualization in a wide range of biomedical and material applications without the requirement for labeling of a specific cellular entity, sectioning, or clearing.

Hard X‐ray tomography with (sub)micrometer resolution has been demonstrated in several areas of neuroimaging.^4^, ^13^ However, reaching an isotropic spatial resolution of less than 100 nm for physically soft tissues without applying a contrast agent remains a challenge.^12^ Phase‐contrast imaging is particularly promising in this regard.^14^ In phase imaging, the contrast is given by the phase shift induced by the sample. The reconstructed quantity is usually the distribution of the refractive index decrement δ(*x*,*y*,*z*). In the hard X‐ray range, for soft materials, mainly consisting of low atomic number elements, the magnitude of the refractive index decrement δ can be three orders of magnitude higher than the imaginary part β of the refractive index, which is related to attenuation contrast. There are many methodologies for phase‐contrast imaging but all transform phase shifts caused by the sample into an intensity modulation that is recorded by the detector.^15^ Propagation‐based phase‐contrast imaging and its most prominent application—holotomography are particularly acknowledged for quantitative high‐resolution imaging.^16^ The term holotomography, introduced in 1999, relates to the in‐line Gabor holography.[[qv: 16b,17]] In holotomography, which originates from the combination of holographic and tomographic reconstruction, one recovers the real and imaginary part of the refractive index. In‐line holography uses a partially coherent beam that interferes with itself, so that no separate reference beam is required. In other words, the interference occurs between the nonscattered wave (direct beam) and the scattered beams. Hence, in‐line holography is relatively simple by today's standards, but actually the original and oldest, holography method.

Herein, we report on investigations into human brain nanoanatomy with synchrotron radiation‐based phase‐contrast hard X‐ray nanoholotomography (XNH) at the ID16A‐NI nanoimaging beamline (European Synchrotron Radiation Facility (ESRF), Grenoble, France),^18^ with isotropic voxel sizes down to 25 nm. As a proof‐of‐principle demonstration, we recorded XNH data for human cerebellum and neocortex tissues embedded in a standard histology paraffin/plastic polymer mixture, JB‐4 Plus and epoxy resin in which well‐defined cellular and subcellular structures of Purkinje, granule, stellate, and pyramidal cells are visualized. The presented segmentations of cellular and subcellular structures, including cell soma, nucleus, nuclear envelope, and nucleolus, enable the assessment of nanoscale components. We also noted that XNH measurements do not impair routine histopathological examination. To demonstrate the validity, we compare the virtual sections obtained by XNH with corresponding histological hematoxylin and eosin (H&E) stained sections, demonstrating the accurate correlation. These results suggest that XNH can expand the standard histopathological examination from 2D to 3D. Hence XNH can be foreseen as a powerful, complementary technique for exploiting the wealth of subcellular brain morphology in thick samples (up to ≈600 µm).

## 2 Results

### 2.1 Nanoholotomography for Neuroimaging

**Figure 1:**
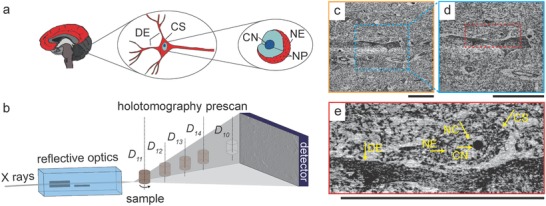
a) Schematic representation of the hierarchical organization of human brain. b) Schematic representation of XNH experimental setup used for holotomography and single‐distance measurements— prescans. c–e) Based on a single‐distance (*D*_10_) scan, the part with a selected pyramidal neuron was measured with effective pixel sizes of c) 100 nm and d) 50 nm at four propagation distances (*D*_11_, *D*_12_, *D*_13_, and *D*_14_). The data with an effective pixel size of e) 50 nm binned twice enable the discrimination of cellular and subcellular structures: cell soma (CS), nuclear envelope (NE) enclosing the nucleolus (CN) and structures within nucleus (NC). The dendrite of an adjacent cell (DE) is readily distinguishable. Scale bars correspond to 50 µm.

As limitations given by wavelength are shifted to subnanometers, application of X‐ray optics can extend spatial resolution far into the nanometer range allowing visualization down to the subcellular level (**Figure** **1**a,e). Propagation‐based phase‐contrast imaging can be performed in the holotomography configuration, where images are recorded at several object‐to‐detector distances, and single‐distance configuration, where only one propagation distance is used. The phase contrast is obtained by leaving the X rays propagate after the interactions with the tissue. The detector, placed ≈1.2 m downstream from the specimen, records the interference patterns at the selected sample‐detector distances. The variation between the farthest and closest distance is on the order of 0.03 m. The recorded data follow the Fresnel diffraction model, which is used to retrieve the phase shift induced by the tissue and ultimately, by combination with tomography, one can reconstruct the 3D electron density distribution in the tissue of interest. For our multiscale approach, first a fast overview (reconstructed volume is a cylinder of 400 µm height and 400 µm in diameter) scan with lower spatial resolution (effective detector pixel length of 200 nm and consequently a voxel size of the reconstructed data set of 200 × 200 × 200 nm^3^) was performed. This prescan enabled us to select regions to image in a holotomography configuration at four propagation distances with voxel lengths ranging from 130 to 25 nm (Figure 1b). In one of the formalin‐fixed paraffin‐embedded samples, this region contained a pyramidal neuron, which we subsequently imaged with effective voxel length of 100 nm (Figure 1c) and 50 nm (Figure 1d). Overall, the data were collected with effective voxel sizes of 200, 130, 100, 50, and 25 nm, and related spatial and density resolutions for visualizing the neuronal architecture were determined.

In the single‐distance configuration, we have qualitatively evaluated the imaging results for the human cerebellum and cortex samples where several preparation and embedding steps were carried out, as described by Müller and co‐workers^19^: formalin‐ or ethanol‐fixed samples embedded in JB‐4 Plus (JB‐4 Plus embedding kit, Polysciences Inc), formalin‐fixed dehydrated samples embedded in epoxy resin with or without unspecific staining by osmium tetroxide. It was observed that the contrast for the formalin‐fixed not dehydrated specimens is insufficient to identify individual neurons, while formalin‐fixed dehydrated specimens embedded in JB‐4 Plus and epoxy resin provided comparable results when features of typical neuron dimensions can be discriminated. Application of the staining protocol for cell identification^19^ resulted in motion artefacts, potentially due to thermal expansion, and nanosized contaminants. Thus, similar to the absorption‐contrast tomography,^20^ formalin‐fixed paraffin‐embedded specimens provide improved data quality. Therefore, the imaging experiments were performed with formalin‐fixed paraffin‐embedded specimens.

For the formalin‐fixed paraffin‐embedded neocortex specimen, the data recorded with a pixel size of 50 nm and binned by a factor of two (Figure 1e), in order to increase the density contrast at the expense of spatial resolution,^21^ exhibit a contrast‐to‐noise ratio (CNR) with respect to the background of 0.806 [0.795 0.818], whereas the CNR of the data with 100 nm voxels corresponds to 0.734 [0.731 0.762]. The associated spatial resolutions are 160 [74 243] nm and 305 [260 369] nm, respectively. These quantitative metrics are related directly to the medical relevance of the acquired data. While the nuclear boundary, compartmentalizing nuclear content, can be distinguished in data acquired with both pixel sizes, namely 50 nm with a binning factor of two and 100 nm, the accurate estimation of envelope thickness is only possible based on the data with an effective voxel length of 50 nm. In both datasets, plasma membranes bounding the cell can hardly be discriminated. Nevertheless, one can identify the cell boundary as the refractive index of the cell sufficiently differs from the one of the surrounding neuropil.

**Figure 2:**
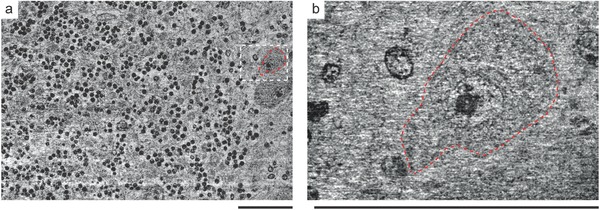
Based on the scan with an effective pixel size of a) 130 nm, a region containing a Purkinje cell (red‐colored dashed line) was identified and scanned with an effective pixel size of b) 25 nm. Scale bars correspond to a length of 50 µm.

To demonstrate the resolution capabilities of XNH for subcellular structures, we scanned a human cerebellum tissue sample with an isotropic pixel size down to 25 nm. Based on a scan with an effective pixel size of 130 nm (**Figure** **2**a), we selected a region containing a Purkinje cell. Figure 2b represents tomography data recorded with an effective voxel length of 25 nm. Due to the low CNR of 0.260 [0.249 0.272] provided, the normalized modulation transfer function (nMTF) was performed by taking the median over ten slices,^22^ resulting in an upper estimation of the spatial resolution of 88 [60 159] nm. Despite the decrease in CNR, an improvement in spatial resolution is noticeable and subcellular structures, including the nuclear envelope and the nuclear pores, are resolvable. The XNH data acquired with an effective pixel size of 25 nm showcase the ability to discriminate between the nucleus represented by the lower electron density and the cytoplasm exhibiting a higher electron density. The region between the nucleus and the cytoplasm of the Purkinje cell shows an almost spherical shape and an inhomogeneous electron density distribution. Its size and location corresponds to the nuclear boundary. In addition, one finds a nanostructure of higher electron density near the nucleolus. This nanostructure potentially arises from the perinucleolar chromatin.

### 2.2 One‐to‐One Correlation with Histology

**Figure 3:**
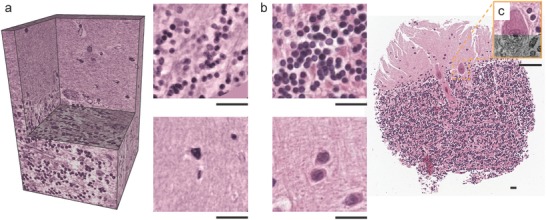
Tomography volume visualized in the a) RGB color map in order to resemble the b) H&E‐stained histological section. For comparison of granule (top) and stellate cells (bottom) XNH data were median‐filtered over 20 slices. c) Combined image showcases the superior resolution of XNH compared to conventional histology. Scale bars correspond to a length of 20 µm.

3D XNH data contains comprehensive morphological information and can be displayed in grayscale (Figure 2) or false color (**Figure** **3**a). To demonstrate the performance of the imaging modality, we compare the virtual sections obtained by XNH with corresponding histological sections stained with H&E—gold standard for the examination of tissue biopsies.^23^ To mimic the H&E staining, the XNH data acquired with an effective pixel size of 100 nm was converted from grayscale to the red‐green‐blue color model (RGB) space, by using the inverse transformation, that is, RGB to grayscale, of the H&E‐stained histology section (Figure 3b). For comparison, magnifications emphasize cellular structures, that is, granule (top) and stellate cells (bottom). Although XNH data showcase the superior to light microscopy resolution (Figure 3c), their correlation is important, linking the tomographic observations to the histological features used in diagnostics.

### 2.3 Rebuilding Neuronal Architecture

The difference in the refractive index of the cell and the surrounding neuropil enables us to segment individual cells and to derive the related quantities. The contrast of our XNH data was sufficiently high for expert‐based examination, yet insufficient for simple automated segmentation approaches. In the current study, even adaptive thresholding fails to segment individual pyramidal neurons, since the refractive index of the cells does not differ enough from background values, that is, the surrounding neuropil. Interactive learning and segmentation, such as ilastik,^24^ have been applied successfully to segment components in electron microscopic images. This software, however, fails to segment cells in the present case, because in microscopy data, the structures of interest are labeled, thus providing high contrast with respect to the background. As a consequence, we developed a two‐step framework for the fully automated segmentation of cortical pyramidal neurons. The structure rests upon a recently designed procedure that is combined with a sparse field method (SFM) of active contours implemented using level sets.[[qv: 13b,25]] In order to verify the automated procedure, we performed semiautomated segmentation using the Image Segmenter app implemented in MATLAB (2016a, The MathWorks, Inc., Natick, Massachusetts, USA), in combination with an SFM. Here, pyramidal neurons were visually identified and individually annotated in a single slice by Image Segmenter. The SFM algorithm automatically extended these seeds up to the entire volume.

**Figure 4:**
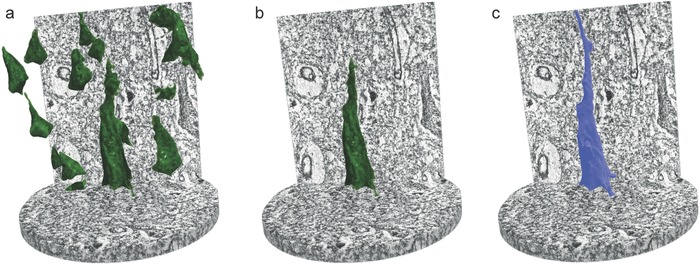
Automated segmentation of pyramidal neurons within a neocortex block, measured with an effective pixel size of 50 nm and a field of view with 100 µm height and 100 µm in diameter. To verify a,b) automated segmentation, c) semiautomated segmentation was performed.

Using the neocortex specimen measured with an effective pixel size of 50 nm binned with a factor of four, we demonstrate the capabilities of our fully automated segmentation strategy for pyramidal neurons (**Figure** **4**a): cells with a somata size within the specified range, not touching the sample border, were successfully segmented by automated (Figure 4a,b) and semiautomated (Figure 4c) approaches, resulting in an automated method sensitivity of about 90%.

### 2.4 Segmenting Subcellular Structures

The data acquired not only assist in identifying individual neurons, but also provide sufficient resolution for discriminating nanostructures including cell soma, nuclear boundary, nucleus, and nucleolus. Furthermore, these structures have a distinctively higher electron density than surrounding cytoplasm, in which case an intensity‐based, region‐growing segmentation framework implemented in the commercially available software package VGStudio MAX 2.0 (Volume Graphics, Heidelberg, Germany) is sufficient.

**Figure 5:**
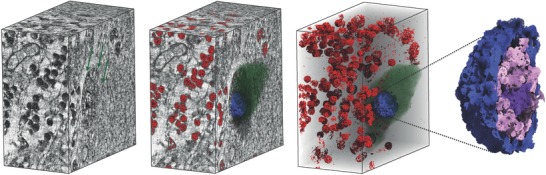
A 3D rendering of (sub)cellular structures within a Purkinje cell, measured with an effective pixel size of 100 nm. Region‐growing segmentation allows for the extraction of cell soma (green), nuclear envelope (blue), nuclear content (pink), and nucleolus (violet). Intensity thresholding (red) enables the discrimination of granule cell nuclei. Average diameter of Purkinje cell soma: 54 µm; average diameter of Purkinje cell nucleolus 3.5 µm.

**Figure** **5** displays a 3D rendering of (sub)cellular components within a Purkinje cell, using data with a voxel length of 100 nm. Region‐growing segmentation allows for the exclusion of cell soma, nuclear boundary, and structures within nucleus, while intensity thresholding helps differentiate between granule cell nuclei. We have observed Purkinje cells as large, pear‐shaped neurons with an average soma diameter of 50 µm and an envelope curvature of 38 µm.

## 3 Discussion

Visualization plays an important role in medical research, as there is a direct correlation between abnormalities in size, shape, or topology of neurons and brain disorders. For example, many pathological brain conditions are associated with cell loss,^26^ abnormal cellular, or dendritic morphology.^27^ Similarly, changes at the subcellular level have been reported for neurodegenerative disorders, for example, membrane damage inducing curvature adaptation,^28^ axon demyelination, and abnormal morphology of microglia in Alzheimer's disease. These pathological (sub)cellular changes are within the resolution range of XNH. The presented segmentations of cellular and subcellular structures can provide quantitative measures, for example, volume or dimension values of subcellular structures, cell number, or shapes. For example, the soma diameter and the envelope curvature of the Purkinje cells were calculated.

The resolved subcellular structure should be reinterpreted to be assigned to specific organelles based on the comparison with clinically approved modality. For this study, virtual XNH slices were compared to the 2D H&E‐stained histology sections. H&E staining, however, frequently used in histopathological investigations, is not the most powerful approach. For example, it is not optimized for the visualization of individual Purkinje cells with subcellular details. It was previously assumed that poor detectability of subcellular structures in H&E‐stained histological sections is related to postmortem autolysis.[[qv: 13b]] The XNH data acquired allow for the identification of anatomical micro‐ and nanostructures, including the nuclear boundary, in agreement with the related 2D histology data. While the comparison of XNH and light microscopy is suboptimal due to the resolution differences, it did confirm that XNH can be used to identify (sub)cellular components. The current goal is to one‐to‐one correlate the modalities to extract clinically useful information for a diagnosis and categorize subcellular objects into individual organelles.

The subcellular structures can be imaged by means of electron microscopy, transmission electron microscopy, or fluorescence microscopy.^29^ For example, high‐resolution, isotropic electron microscopy techniques combined with tissue ablation, such as focused ion beam scanning electron microscopy (FIB‐SEM), reach isotropic voxel sizes of about 4 nm.^30^ The procedure to cover significant tissue volumes, however, is considerably time‐consuming. In contrast, using XNH, one can, for example, image an entire Drosophila brain at 50 nm isotropic voxel size in about 12 h or at 100 nm isotropic voxel size in about 4 h. While the resolving power is not the same as in the case of FIB‐SEM, the technique we propose empowers the biological community to pursue a large spectrum of studies not feasible before.

Moreover, most nanoscopic techniques require sample staining, which will possibly alter the native state, even when using cryopreservation. By using X‐ray phase contrast, one can image both label‐free and labeled samples. The presented label‐free approach has the advantage of no detectable impact on the specimen during the scan, despite the high photon density necessary for the true nanometer resolution. Thus, the same object has been examined multiple times without detecting any modification. For osmium‐stained tissues, however, the absorption‐induced heating leads to the deformation of the tissue during the data acquisition.

The large specimens for XNH can simply be paraffin embedded following the standard well‐established histological protocols directly applicable to clinical specimens. The proposed method, as it was shown during the study, is particularly compatible with archival specimens of formalin‐fixed paraffin‐embedded tissue, which, in large quantities and covering nearly every branch of medicine, is universally available around the globe, allowing retrospective studies of various diseases.^6^ Some of these samples are indeed very precious and imaging them in a nondestructive manner is likely to add value. The nanoholotomography images obtained indicate that the standard preparation procedure to obtain slides after formalin fixation and paraffin embedding gives rise to significant tissue damages, which impede the segmentation of dendrites and axons. As the technique is also compatible with tissue cryopreservation,[[qv: 18b]] one can image nonstained, high‐pressure frozen tissue in near future. However, cryofixation (using high‐pressure freezing) and freeze substitution which least alters the native state are usually limited to samples with thickness less than 200 µm.^31^

**Figure 6:**
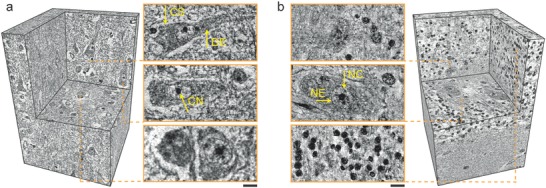
3D volume rendering of cerebellum and neocortex specimens, highlighting electron microscopy‐like data quality acquired with clinically relevant tissue preparation and without cutting. Visualization of a) neocortex and b) cerebellum blocks measured with an effective pixel size of 100 nm. Magnifications of virtual cutting planes illustrate cellular and subcellular features, including cell somata (CS), dendrites (DE), a nuclear envelope (NE), a nucleolus (CN), and structures within nucleus (NC). Scale bars correspond to a length of 10 µm.

Already the use of 100 nm voxels makes it possible to image neocortex (**Figure** **6**a) and cerebellum (Figure 6b) tissues at sufficient resolution to identify cell types. The desired spatial resolution restricts the accessible volume. The local tomography approach, however, allows stitching submillimeter‐size volumes measured separately within the same object, so that currently a significant portion of the human cerebral cortex can be 3D imaged. As the handling of big data advances, the entire cerebral cortex can become available in future.

The methodology allows for the nondestructive imaging, as it does not require physical sample slicing or staining. Thus, no physical changes are introduced to the specimens during the imaging experiment, as opposed to the alternative techniques, such as gold standards for micro‐ and nanostructured investigations, namely light and electron microscopy. This is crucial for many studies with limited specimen availability, for example, studies of rare genetic disorders or rare mutations. Thus, there is a wide range of potential applications where XNH can be used as an effective in vitro approach before applying established complementary imaging techniques.

In contrast to sectional modalities, XNH data can be virtually sectioned at arbitrary orientation and technical difficulties such as perfect continuity between consecutive slices are overcome. For example, the dendritic tree of pyramidal neuron profusely branches varying the orientation. Moreover, cellular and subcellular structures change in shape and dimension over the volume. Thus, for characterization of its alteration conventional 2D microscopy is insufficient, and 3D characterization is necessary.

Even though micro‐ and nanostructures are important components, advancing our understanding of brain functioning requires knowledge of its molecular composition.^32^ However, XNH does not identify molecules or chemicals in the tissue, one added advantage of the modality is the ability to record quantitatively local refractive indices proportional to the local electron density, delivering data as known from quantitative phase imaging, for example, differential interference contrast microscopy,^33^ hard X‐ray grating interferometry,[[qv: 13a]] or 3D refractive index tomography.^34^

XNH has significant advantages over traditional histology while maintaining compatibility with gold standard. Considering the cerebellum, we demonstrate that histology and XNH complement each other, in that histology yields 2D functional information based on a variety of histochemical stains, while tomography reveals quantitative morphological information over the entire tissue volume, with significantly improved spatial resolution.

## 4 Conclusions

We have demonstrated that XNH allows generating 3D images of large portions of brain tissue ex vivo without the need for sectioning, staining, or sample preparation outside clinical practice. Thus, it could find a wide application as an imaging tool in neuroscience research. As a proof‐of‐concept experiment, we showed micro‐ and nanostructural images of human cerebellum and neocortex at multiple resolution scales. This experimental approach paves the way to an automated and objective approach to study brain's nanoanatomy alterations caused by pathological conditions or medical interventions. XNH provides extraordinary capabilities for 3D imaging of cells, in that subcellular structures can be identified in a label‐free and time‐efficient manner with quantitative values related to biochemical properties. Thus, nanoholotomography bridges the spatial resolution gap between optical and electron microscopy while giving access to nanoscale isotropic resolution 3D data of relatively large tissue volumes. As the acquisition rate can be increased by orders of magnitude and as penetration of hard X‐rays through soft tissue is virtually unlimited, full‐scale subcellular mapping of the human brain is within reach.

## 5 Experimental Section

*Specimen Preparation*: Brain specimens were obtained from donated bodies. All donors of the program contributed their bodies or parts of their bodies to education and research purposes. Informed consent for scientific use was obtained in written form and the procedures were conducted in accordance with the Declaration of Helsinki. The brains were fixed in 4% histological‐grade buffered formalin, before samples of the neocortex, adjacent white matter, and cerebellum were excised, dehydrated in ethanol, cleared in xylene, and embedded in a paraffin/plastic polymer mixture (Surgipath Paraplast, Leica Biosystems, Switzerland). Cylindrical specimens with a diameter of 510 µm and a height of ≈1 mm were cut from paraffin blocks using a metal punch.

*Specimen Evaluation*: Before synchrotron radiation–based imaging, the specimens were preliminarily evaluated by means of laboratory‐based absorption‐contrast micro computed tomography system nanotom m (GE Sensing & Inspection Technologies GmbH, Wunstorf, Germany). The focal spot diameter was adjusted to 1 µm and the measurements were performed in the fast scan mode with a total scan time of 12 min, an effective pixel size 1 µm, a tube voltage of 60 kVp, and a beam current of 350 µA.

*Tomography Data Acquisition*: Tomography images were acquired at the ID16A‐NI nanoimaging beamline of the ESRF in Grenoble, France.^18^ The schematic representation of XNH experimental setup used for nanoholotomography and single‐distance measurements is shown in Figure 1b. The high‐brilliance coherent beam could be focused to a spot measuring down to 13 nm. For the imaging experiments, an X‐ray beam with photon energy of 17 keV was focused to a spot of ≈25 nm (horizontal) × 37 nm (vertical) via a pair of multilayer‐coated Kirkpatrick–Baez mirrors for vertical and horizontal focusing. The photon flux was about 3 × 10^11^ photons per second. The sample was mounted on a rotation stage downstream from the focal plane inside a vacuum chamber. The pressure in the vacuum chamber was between 10^−7^ and 10^−8^ mbar. The detector, composed of a scintillator converting X rays to visible light, magnifying optics, and a charge‐coupled device (CCD) camera (FReLoN, ESRF, Grenoble, France) with 4096 × 4096 pixels, was placed ≈1.2 m away from the focal plane. The detector pixels were binned two‐by‐two to an effective size of 3 µm. Four tomographic scans were recorded by placing the sample at preselected distances between the focus and the detector. The divergent beam yielded a geometrical magnification *M* = (*D*_1_ + *D*_2_)/*D*_1_, where *D*_1_ denotes the distance between focal plane and sample, and *D*_2_ denotes the distance between sample and detector. As the samples were larger (≈510 µm) than the field of view, local nanoholotomography measurements were performed after selecting the relevant region on a single‐distance low‐resolution tomography scan—a prescan with an effective pixel size of 200 nm. The summary of experimental parameters is listed in **Table** **1**.

**Table 1:** Scanning parameters. *l*: effective pixel size; *N*: number of projections; *t*: exposure time; number of detected photons per pixel and projection; number of detected photons per pixel during the acquisition of the tomography data; *D*_1_: focus to sample distances

Sample	*l* [nm]	*N*	*t* [s]	Number of photons/(pixel*projection)	Photons/(pixel*scan) [10^6^]	*D*_1_ [mm]
Cerebellum	200	1200	0.25	500	0.6	80.533
	130	1200	0.25	500	2.4	52.346, 54.592, 63.575, 82.226
	50	1200	0.25	500	2.4	20.133, 20.997, 24.452, 31.625
	25	1800	0.25	500	3.6	10.066, 10.499, 12.226, 15.812
Cortex	200	1200	0.30	600	0.7	80.533
	100	2000	0.30	600	4.8	40.266, 41.994, 48.904, 63.251
	50	2000	0.30	600	4.8	20.133, 20.997, 24.452, 31.625
Cerebellum	200	1200	0.30	600	0.7	80.533
	100	1900	0.30	600	4.6	40.266, 41.994, 48.904, 63.251
	50	1900	0.30	600	4.6	20.133, 20.997, 24.452, 31.625

In order to retrieve the phase maps,[[qv: 16b,35]] a set of four radiographs at a given rotation angle was normalized with respect to the incoming beam, then brought to the same magnification, aligned and used in an adapted contrast transfer function algorithm to determine the phase shift. Tomographic reconstructions were obtained by filtered back projections using PyHST2.^36^ The reconstructed images provided the 3D distribution of the real part of the complex refractive index, which is proportional to the local electron density ρ_e_.

*Histology*: Subsequent to tomography, samples were re‐embedded in a standard paraffin block and sectioned at a thickness of 4 µm for histological examination. Sections were mounted on glass slides and stained with H&E. The resulting slides were digitized using a microscope slide scanner (3DHistech Pannoramic MIDI, Sysmex Suisse AG Horgen, Switzerland) with an effective pixel size of 243 nm.

*3D–3D Registration*: For comparison of the datasets measured using the selected voxel lengths, translation registration was performed, using the library provided by insight segmentation and registration toolkit (ITK),^37^ allowing for the selection of the same region of interest for the analysis.

*Quantitative Metrics*: The quantitative comparison was carried out in MATLAB (2016a, The MathWorks, Inc., Natick, Massachusetts, USA). The quantitative evaluation of XNH data was based on the calculation of a volumetric CNR and an edge‐based upper limit of spatial resolution.^22^ CNR was defined as: CNR = ǀ*I*_1_ – *I*_2_ǀ/*√*(σ_1_^2^ + σ_2_^2^), where *I*_1_ and *I*_2_ indicate the mean intensities of homogeneous components within the specimen, and σ_1_ and σ_2_ are corresponding to the standard deviations. The intensity histograms were fitted with two Gaussians to extract *I* and σ.^38^ To estimate the upper limit of spatial resolution, the intersection of the nMTF was used with the 10% value. For the calculation, a region was chosen at the nucleus–nuclear–plasma interface. In order to perform an accurate comparison of the data measured with the selected effective pixel sizes, 50 nm pixel size data were binned with a factor of 2, using the library provided by ITK.^37^ Due to the low CNR provided by the data acquired with 25 nm pixel size, the nMTF was performed taking the median over ten slices resulting in an upper estimation of the spatial resolution. For the estimation of Purkinje neuron curvature, it was assumed that the cells are elliptical. Curvature was defined as: *R* = *a* ^2^/*b*, where 2*a* indicated the major axis of the ellipse and 2*b* the minor axis of the ellipse.

*Data Segmentation*: For the automated segmentation of pyramidal neurons within the neocortex block, a two‐step framework was used. An approach was also applied that had already been successfully applied to a large numb er of Purkinje cells in the human cerebellum in combination with SFMs of active contours using level sets.[[qv: 13b,25]] The first step was based on feature‐based Frangi filtering that detected structures of interest by analyzing the eigenvalues of the 3D Hessian matrix. The parameters were chosen as α = 0.5, β = 0.1, and γ = 60, which accounted well for high‐intensity changes on the margins of the cells but neglected strong noise in the background, due to the high value for parameter γ. The filter parameters also included the radius range of 15–25 voxels with a step size of *r* = 2. The filter result was binarized using a threshold of 0.01. Objects were neglected if they were smaller than 20 000 voxels or only partially segmented on the margin. The segmentation results of the Frangi filter were used as an initialization mask for the SFM. Segmentation started with images partitioning in a slice‐wise manner with *N*_it_ = 900 iterations and the relative weighting of curve smoothness of ζ = 0.01, followed by a 3D step with *N*_it_ = 60 and ζ = 1, where the output of the 2D steps was used as an initialization mask. All parameters were selected based on visual inspection to avoid noise segmentation. Objects smaller than 300 voxels were neglected. In order to verify the results of the automated approach, semiautomated segmentation was performed, whereby each pyramidal neuron was visually identified and individually annotated in one selected slice by means of an Image Segmenter app implemented in MATLAB. This segmentation was used to initialize 2D SFM segmentation in an iterative manner. The subsequent steps were equivalent to the automated approach. For the estimation of the statistical performance of the automated segmentation approach, sensitivity metric (*S*) was used. The metric describes to the probability of correct detection and is defined as the ratio between the correctly detected objects (TP) and the total number of detected objects (*T* ): *S* = TP = *T*. Subcellular structures were semiautomated segmented using an intensity‐based, region‐growing segmentation framework with multiple seed points implemented in the commercially available software package VGStudio MAX 2.0 (Volume Graphics, Heidelberg, Germany).

## Conflict of Interest

The authors declare no conflict of interest.
